# Do intra-articular hyaluronic acid injections delay total knee replacement in patients with osteoarthritis – A Cox model analysis

**DOI:** 10.1371/journal.pone.0187227

**Published:** 2017-11-20

**Authors:** Angélique Delbarre, Bernard Amor, Isabelle Bardoulat, Aymeric Tetafort, Nathalie Pelletier-Fleury

**Affiliations:** 1 INSERM, Center for research in Epidemiology and Population Health, Université Paris-Saclay, Université Paris-Sud, UVSQ, Villejuif, France; 2 Université Paris Descartes, Paris, France; 3 QuintilesIMS, Paris La Défense 2, France; Illinois Institute of Technology, UNITED STATES

## Abstract

Due to the growing worldwide prevalence of knee osteoarthritis, the optimal management of this issue is critical for reducing its burden.

**Objectives:**

This study aimed to describe patients treated for knee osteoarthritis between 2006 and 2013 in France and to compare the delay from diagnosis to total knee replacement between patients who received intra-articular hyaluronic acid injections and those who did not receive the injections. A second objective was to compare direct medical costs for ambulatory care between treatment groups.

**Materials and methods:**

Patients were selected from a representative sample of the real world administrative claims database using an algorithm developed by experts from the scientific committee of the study. Data were matched with the medico-administrative database for hospital care. A Cox proportional hazards model was stratified for the treatment group and adjusted for available socio-demographic and medical covariates to compare restricted mean survival times at different time points (1, 3, 5 and 7.5 years) between groups. Costs were expressed in 2013 euros.

**Results:**

A total of 14,782 patients were treated for knee osteoarthritis (67% women; mean age = 68 years). Among this population, 1,662 patients had total knee replacement (11.2%). At each time point, restricted mean survival time without total knee replacement was significantly higher (p-values<0.001) for hyaluronic acid group, from +51 to +217 days at 1 and 7.5 years, respectively. For the year preceding total knee replacement, the means for total direct medical costs were similar between groups, €744 vs €805 for treatment and control groups, respectively, (p-value = 0.104). Intra-articular injections accounted for less than 10% of the total costs.

**Conclusion:**

This is the first retrospective longitudinal study involving knee osteoarthritis patients using medico-administrative databases in France. The results support the effectiveness of hyaluronic acid injections in delaying total knee replacement and show that patients treated with hyaluronic acid have similar direct medical costs for ambulatory care compared to patients treated with corticosteroids only.

## Introduction

Osteoarthritis (OA) of the knee is a major public health problem, and its optimal management is critical to reducing the burden of illness in developed countries [1,2]. According to the World Health Organization, osteoarthritis is among the ten most disabling chronic diseases in developed countries [1]. The knee is the most commonly affected joint in the human body [3]. In France, approximately 4.7% of men and 6.6% of women between 40 and 75 years of age have symptomatic OA of the knee, which causes pain and affects activities of daily living [4,5]. The prevalence of knee OA is higher in women. There is also an increased risk of occurrence of OA with age, obesity and in people involved in certain manual occupations [6–9]. In the early 2000s, OA, including knee OA, accounted for approximately 1.7% of the French Health Insurance’s annual expenditure (1.6 billion Euros). Hospital costs accounted for approximately 50% of the total cost [10]. Similar figures were found in other countries [11–13].

The management of knee OA is aimed at reducing pain and disability using non-pharmacological and pharmacological treatments. A combination of education, exercise and treatment such as analgesics or non-steroidal anti-inflammatory drugs (NSAIDs) is recommended for mild-to-moderate knee OA [14]. As the disease progresses, intra-articular (IA) corticosteroid (CS) injections may also be used as local treatment to relieve pain. However, long-term use of CSs is not recommended, especially for patients with comorbidities such as hypertension and diabetes [15]. IA hyaluronic acid (HA) injections have been developed and widely used over the last 20 years. By increasing synovial fluid, which protects the knee joint, IA HA can reduce pain for several months, while the effect of IA CS on pain is immediate but shorter in duration [16,17]. These injections might also delay total knee replacement (TKR), which is the last resort treatment for severe knee OA with structural changes [18,19]. In France, the TKR rate increased by 90% between 2000 and 2013, compared to 50% on average in the other Organization for Economic Co-operation and Development (OECD) countries [20]. TKR usually allows the patient to recover normal function of his or her knee [20,21]; however there may be surgical complications and patients may require subsequent procedures to correct or improve the defects [19,22]. The risk of revision surgery is estimated to be between 5% and 12% ten years after initial TKR [23,24]. Moreover, TKR is associated with substantial surgery and post-surgery costs [25]. According to Le Pen et al., between 2001 and 2003, more than 38,000 TKR were performed each year with an average cost per surgery of €4,500 [10]. However, this evaluation did not take into account the cost of the knee prosthetic device. In a more recent French Health Insurance report, the average global cost of TKR was estimated at €8,715 in the public sector and at €7,594 in the private sector [26]. In addition, approximately 20% of the annual hospital costs for OA, including knee OA, were related to postoperative care and rehabilitation. Therefore, it is crucial that patients with knee OA maintain mobility of the knee as long as possible to delay or avoid TKR.

As previously stated, IA HA injections have been used as an alternative or as an adjunct to conventional treatment, albeit differing conclusions on its effectiveness [27–30]. The debate essentially concerns periods of the use of IA HA injections in knee OA management. Certain authors have recommended limiting the use of HA to those patients presenting an inadequate response to initial therapy such as NSAIDs or IA CS injections [31]. Conversely, several studies concluded that HA should be used irrespective of knee OA severity (mild, moderate, severe) given that the treatment had a positive global assessment and was well-tolerated by patients [32,33]. The National Authority for Health in France, the Haute Autorité de Santé (HAS), currently defines HA as a treatment option, more specifically for patients who are intolerant to and/or have an inadequate response to NSAIDs. Some studies have shown that IA HA injections can delay the need for TKR in some patients [34–36]. According to these studies, the use of HA might delay surgical treatment up to 2 years, depending on the number of IA HA injections [19,37,38]. However, most of these studies were short-term clinical trials with some using simulation methods such as a discrete event simulation model, while others did not. In addition, only few recent observational studies in the US have analyzed the association between HA and time-to-TKR [37–40].

In this context, the present study aimed to describe the characteristics of patients treated for knee OA between 2006 and 2013 using a real-world administrative claims database, the Échantillon Généraliste des Bénéficiaires (EGB) and to compare the time from diagnosis to TKR between patients receiving IA HA injections and those not receiving IA HA injections over the same time period. A second objective was to perform a cost analysis from the payer perspective comparing direct medical costs for ambulatory care between treatment groups before TKR.

## Materials and methods

Two databases were used in this study: the EGB and a health administrative hospital discharge database for medical, surgical and obstetrics wards and the Programme de Médicalisation des Systèmes d’Information–Médecine Chirurgie Obstétrique (PMSI-MCO) databases. The EGB, which has been available since early 2006, boasts a representative sample composed of 1/97^th^ of a larger database, the Système National d’Information Inter-Régimes de l’Assurance Maladie (SNIIRAM), which is a health administrative database that provides exhaustive information on all reimbursed ambulatory care of people who are covered by any of the main French health insurance funds (98% of the population). More than 700,000 patients are included in the EGB for whom outpatient procedures, laboratory tests, physician visits and drug dispensing claims are available along with the specialty of the prescriber among other data. This database also includes information on patient demographics (gender, date of birth, date of death if applicable, geographical location) and declared chronic diseases that are entirely covered by health insurance, which refers to them as long-term diseases (LTD). Of note, this list does not contain knee OA.

Data related to private or public hospital stays were collected from the national PMSI-MCO database. This is a health administrative hospital discharge database set up in the early 1990s. This database provides exhaustive information on hospital care in France such as diagnoses (coded by the physician using the International Classification of Diseases, 10th revision [ICD-10]), surgical procedures performed (including TKR), underlying comorbidities and possible complications, length and costs of stays, etc. Each patient's stay is classified by the Diagnosis Related Group (DRG) according to the information documented by the physician. According to the Classification Commune des Actes Médicaux (CCAM), the classification of medical procedures in the SNIIRAM, surgical stays with initial TKR were identified with the codes from NFKA006 through NFKA009.

For each patient included in the study during the 2006–2013 period, data from EGB and PMSI were linked using a common anonymous patient identifier to explore the associations between the characteristics of knee OA patients, ambulatory care resource utilization including IA injections, and TKR. It should be noted that it was not possible to distinguish which knee was in question regarding injections and TKR when both knees were affected by OA in the same patient.

Access to the databases was subject to prior training and authorization and was approved by the Independent Data Protection Administrative Authority (Commission Nationale Informatique et Libertés, CNIL). Written informed consent was not required as data were anonymized.

### Study population

#### Inclusion criteria

All patients aged 50 years or older and treated for advanced knee OA between 2006 and 2013, both TKR and non-TKR patients, were included in the study. It is important to mention here that the EGB does not contain any clinical information or lab test or X-ray results. Therefore, we developed an algorithm to assist with the selection of these patients. According to the expert opinion of the scientific committee of the study, knee OA patients were likely to meet the following inclusion criteria:

1. At least one X-ray or arthroscopy of the knee followed by an IA injection (CS or HA (S1 and S2 Tables)) during the following 12-month period;
2. Fifty years of age or older at the time of X-ray/arthroscopy of the knee;
3. The injection (CS or HA) was prescribed by a rheumatologist, an orthopedic surgeon, a physician specializing in physical medicine and rehabilitation or a general practitioner (GP).

In France, IA HA injections must be prescribed by a rheumatologist, orthopedic surgeon or physician specialized in physical medicine and rehabilitation. However, situations such as treatment renewals or medically underserved areas might lead to GP’s prescription, which is the reason we took into account IA HA injections prescribed by GPs during the study period.

This IA injection was considered to be the patient’s first IA injection. The X-ray/arthroscopy of the knee was used as a proxy to date the diagnosis of knee OA.

#### Exclusion criteria

Patients who met any of the following criteria were excluded:

1. Death during the first month after the index date, i.e., the diagnosis (proxy defined above);
2. The presence of a gap in the data of at least one year during the follow-up period;
3. Unusual health events such as paraplegia or already having two TKRs.

### Variables

#### Dependent variable and variable of interest

The dependent variable was the length of the follow-up period, i.e., the number of days from diagnosis to the end of follow-up. The end of follow-up is defined as the first following event occurring before the end of the study (12/31/2013). There were four types of end-of-follow-up, three of which were censored (i.e., the event of interest, TKR, did not occur during the time the patient was observed), as follows:

- Having a TKR;
- Death (censor);
- Exit (censor): exit from the national health insurance scheme;
- End of the study period (censor): no other event is observed.

The variable of interest was the treatment group, which was coded “HA” if the patient had at least one IA HA injection in the knee during the follow-up period and “N-HA” if the patient had only IA CS injection(s). Patients who had the two types of IA injections (HA and CS) were considered part of the HA group.

#### Adjustment variables

Three types of adjustment variables were considered as follows:

- Socio-demographics: gender, age at diagnosis and age at TKR;
- General health conditions:
  1. Presence of at least one LTD at diagnosis irrespective of severity (Y/N);
  2. An overall comorbidity score quantified using the Charlson index to reflect the cumulative increased likelihood of one-year mortality (with the following split: 0/1-2/≥3) [41,42];
  3. Mean annual number of visits to the GP during the follow-up period (≤12/13-24/>24);
  4. At least one hospitalization during the follow-up period, excluding TKR (Y/N);
- Knee OA conditions:
  1. Initial prescriber’s specialty ((rheumatology, physical medicine and rehabilitation, orthopedic surgery, GP);
  2. Mean annual number of visits to physicians specializing in rheumatology, orthopedic surgery or physical medicine and rehabilitation (≤4/5-10/>10);
  3. Number of knees with OA (two/one or unknown).

#### Direct medical costs variables

Only direct medical costs in ambulatory care before TKR were considered, i.e., costs of doctors’ visits, drugs and other ambulatory care (see list below) if the care was likely to be related to knee OA and if it was performed (for visits) or prescribed (for other ambulatory care) by a rheumatologist, orthopedic surgeon, physician specializing in physical medicine and rehabilitation or a GP. The direct medical costs that were included were as follows:

- Drug therapies (ATC classes):
  1. A02 (drugs for acid disorders)
  2. D07 (corticosteroids, dermatological preparations)
  3. H02 excluding IA CS injections (corticosteroids for systemic use)
  4. M01 (anti-inflammatory and anti-rheumatic products)
  5. M02 (topical products for joint and muscular pain)
  6. M09 excluding IA HA injections (other drugs for disorders of the musculo-skeletal system)
  7. N02 (analgesics);
- IA CS and HA injections
- Medical devices: canes, crutches, walkers, elastic compression bandages and orthopedic corrections;
- X-rays and arthroscopies of the knee;
- Physical therapies;
- Thermal treatment;
- Visits to doctors:
  1. Rheumatologists, orthopedic surgeons and physicians specializing in physical medicine and rehabilitation
  2. GPs when visits were associated with OA-related prescriptions (see list above: drugs, IA injections, devices, etc.)
- Transportation costs if the date corresponds with a visit to a doctor defined above.

Costs were calculated in € and determined from the health insurance point of view. Costs were relative to 2013. Total ambulatory costs per year were estimated. For the last year before TKR, we also estimated the share of total ambulatory costs.

### Statistical analyses

#### Descriptive analysis

To highlight differences between groups (HA versus N-HA), a descriptive analysis was first performed in the total population of patients treated for knee OA, followed by patients with TKR. For quantitative variables, means and, standard deviations (SD) were computed; for qualitative variables, quartiles and percentages were computed. Different tests were used according to variable distribution; the t-test or Wilcoxon test to compare quantitative variables, and the chi-square test to compare qualitative variables. Tests were bilateral, and a p-value less than 0.05 was considered as significant. Missing data were not replaced because they were expected to be random and were very sporadic. Similar tests were performed to compare ambulatory care costs between groups.

#### Survival analysis

Survival analyses were performed to compare the length of the follow-up period, i.e., time-to-TKR, between treatment groups in the subsample of patients with TKR. As a first step, Kaplan-Meier (KM) curves and a log-rank test were computed. As a second step, a Cox proportional hazards model was used to adjust for covariates. It was stratified for the treatment group variable (HA/N-HA) because the treatment group variable did not match the proportional hazard hypothesis [43]. Restricted mean survival times (RMST) and Zucker test were used to estimate and compare mean survival times of treatment groups at different time points after diagnosis [44–46]. These time points were set before starting analysis and took the maximal length of follow-up into account: 365 days (1 year), 1,095 days (3 years), 1,825 days (5 years) and 2,794 days (7.5 years). We managed this step using the function developed by Zhang (2013) [47]. Confidence intervals of 95% were computed for all parameters.

All analyses were performed using SAS 9.4 (SAS Institute Inc., Cary, North Carolina).

## Results

### Study population treated for knee OA

**Fig 1:**
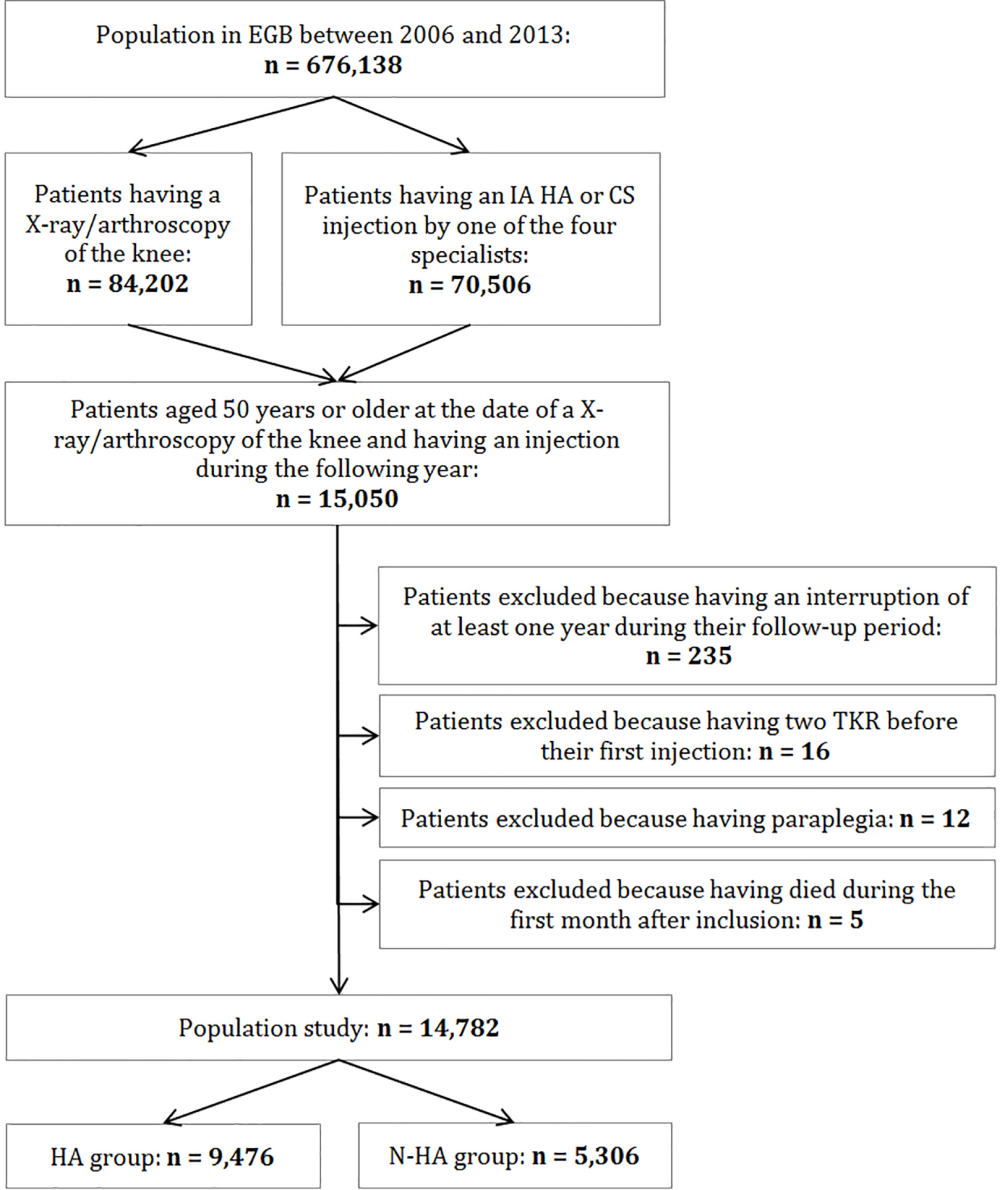
Flow-chart of patients’ selection. IA: intra-articular, HA: hyaluronic acid, CS: corticosteroids, TKR: total knee replacement. HA group: patients who received at least one IA HA injection during their follow-up period, N-HA group: patients who received only CS as IA injections. The four specialists are rheumatologist, orthopedic surgeon, physical medicine and rehabilitation practitioner and general practitioner.

Based on the inclusion criteria, the study population consisted of 14,782 patients aged 50 years or older who had knee OA (Fig 1). The annual incidence rates of knee OA for men and women were estimated to be 0.52% and 0.92%, respectively. In 2013, prevalence estimates for men and women were 3.9% and 6.9%, respectively. Among these 14,782 patients, 1,662 had TKR before December 31^st^, 2013. The prevalence estimate of TKR was 11.2% at that time.

Table 1 summarizes the main characteristics of the study population (n = 14,782). Approximately 67% of patients were women, and the mean age was 68 (±10) years old. The patients were followed for 1,267 (±791) days on average, i.e., approximately 3.5 (±2.2) years. Among them 13,120 (89%) were censored due to the absence of the outcome of interest. Regarding treatment groups, 5,306 patients (36%) had IA CS injections only (N-HA group), while 9,476 patients (64%) had at least one IA HA injection (HA group) during the follow-up period. In the latter group, the first IA HA injection was delivered 182 (±330) days after inclusion on average, and 66% of these patients also received IA CS injections. OA management was significantly different between treatment groups. The first prescription of IA injection by a rheumatologist or a physical medicine and rehabilitation practitioner was more common in the HA group (76% of patients) than in the N-HA group (57%). The mean number of annual visits to specialized physicians was significantly higher in the HA group; that is, HA patients visited joint disease specialists on average 2.1 times more than N-HA patients.

**Table 1:** Descriptive analysis of patients with knee OA.

Characteristic n (%)*	HA group (n = 9,476)	N-HA group (n = 5,306)	All patients (n = 14,782)		P-value**
**Gender**				**0.056**	
Male	3,213 (33.91)	1,717 (32.36)	4,930 (33.35)		** **
Female	6,263 (66.09)	3,589 (67.64)	9,852 (66.65)		** **
**Age at diagnosis **				**<0.0001**	
Mean (±SD)	67.90 (±10.15)	67.26 (±10.85)	67.67 (±10.41)		** **
Median [Q1—Q3]	67 [60–76]	66 [58–76]	67 [59–76]		** **
**Presence of long-term disease(s) at diagnosis **				**0.702**	
Yes	3,228 (34.07)	1,824 (34.38)	5,052 (34.18)		** **
No	6,248 (65.93)	3,482 (65.62)	9,730 (65.82)		** **
**Charlson index **				**0.146**	
0	3,533 (37.28)	1,901 (35.83)	5,434 (36.76)		** **
1–2	4,075 (43.00)	2,364 (44.55)	6,439 (43.56)		** **
≥ 3	1,868 (19.71)	1,041 (19.62)	2,909 (19.68)		** **
**Primo-prescriber's specialty**				**<0.0001**	
General practice (GP)	1,540 (16.25)	2,013 (37.94)	3,553 (24.04)		** **
Rheumatology/Physical medicine and rehabilitation	7,223 (76.22)	3,003 (56.60)	10,226 (69.18)		** **
Orthopedic surgery	713 (7.52)	290 (5.47)	1,003 (6.79)		** **
**Number of annual visits to the GP**				**<0.0001**	
Mean (±SD)	8.32 (±5.70)	9.05 (±6.76)	8.58 (±6.11)		** **
Median [Q1—Q3]	7.11 [4.68–10.79]	7.47 [4.90–11.70]	7.24 [4.74–11.11]		** **
**At least one hospitalization (TKR excluded) **				**0.777**	
Yes	6,388 (67.41)	3,589 (67.64)	9,977 (67.49)		** **
No	3,088 (32.59)	1,717 (32.36)	4,805 (32.51)		** **
**Number of annual visits to the specialists of joint diseases** **†**				**<0.0001**	
Mean (±SD)	3.36 (±3.50)	1.59 (±2.61)	2.72 (±3.32)		** **
Median [Q1—Q3]	2.44 [1.29–4.29]	0.78 [0.20–1.94]	1.81 [0.73–3.61]		** **
**Number of annual IA HA injections** **‡**				**<0.0001**	
Mean (±SD)	2.88 (±5.77)	0 (±0)	1.84 (±4.82)		** **
Median [Q1—Q3]	1.76 [0.93–3.27]	0 [0–0]	0.85 [0.00–2.30]		** **
**Number of days between inclusion and first IA HA injection(s) **				**NA**	
Mean (±SD)	182 (±330)	-	182 (±330)		
Median [Q1—Q3]	70 [23–193]	-	70 [23–193]		
**Number of annual IA CS injections §**				**<0.0001**	
Mean (±SD)	1.57 (±4.63)	1.64 (±4.52)	1.60 (±4.58)		** **
Median [Q1—Q3]	0.88 [0.45–1.74]	0.76 [0.37–1.63]	0.82 [0.41–1.70]		** **
**Number of days between inclusion and first IA CS injection(s)** **§**				**<0.0001**	
Mean (±SD)	209 (±358)	98 (±105)	158 (±278)		
Median [Q1—Q3]	68 [18–234]	54 [12–161]	61 [15–193]		
**Number of follow-up days**				**<0.0001**	
Mean (±SD)	1,267 (±791)	1,329 (±830)	1,289 (±806)		** **
Median [Q1—Q3]	1,131 [585–1,912]	1,231 [595–2,42]	1,169 [588–1,957]		** **
Range	[7–2,916]	[8–2,912]	[7–2,916]		** **
**Type of follow-up end**				**<0.0001**	
Total knee replacement (TKR)	1,296 (13.68)	366 (6.90)	1,662 (11.24)		** **
Death (censor)	388 (4.09)	323 (6.09)	711 (4.81)		** **
Exit (censor)	50 (0.53)	46 (0.87)	96 (0.65)		** **
End of study (censor)	7,742 (81.70)	4,571 (86.15)	12,313 (83.30)		** **

No missing data

HA group: patients who received at least one IA HA injection during their follow-up period, N-HA group: patients who received only CS as IA injections, GP: general practionner, TKR: total knee replacement, OA: osteoarthritis, IA: intra-articular, HA: hyaluronic acid, CS: corticosteroids

* n (%) unless stated otherwise

** T-test, Wilcoxon (for mean difference) or Chi-squared tests (for percentage difference) (NA: not applicable)

† Rheumatologist, orthopedic surgeon and physical medicine and rehabilitation practitioner

‡ Calculated by combining mono- and multi-injections treatments

§ Calculated on 11,561 Patients who received at least one IA CS injection during their follow-up period (n = 6,255 for HA group, n = 5,306 for N-HA group)

Finally, it is noteworthy that the rate of TKR was two times higher in the HA group compared to that in N-HA group (13.7% vs 6.9%, respectively). This crude result associated with the difference in OA management could suggest that HA patients were likely to be at a more advanced stage of knee OA than N-HA patients, unobserved heterogeneity could affect our results. To reduce sources of bias between groups and to address the high global censor rate (nearly 90%) that could disrupt the RMST estimates, the sample was limited to patients with TKR (N = 1,662) to compute the Cox model.

### Comparison of the time from diagnosis to TKR

The bivariate analysis performed on the 1,662 patients with TKR shows that the mean time from diagnosis to TKR was significantly higher in the HA group than in the N-HA group: 864 (±601) days vs 573 (±548) days, respectively (p-value<0.0001). There were also other significant differences between treatment groups, which are summarized in Table 2.

**Table 2:** Bivariate analysis of knee OA patients who had TKR.

Characteristic n (%)*	HA group (n = 1,296)	N-HA group (n = 366)	P-value**
**Gender**			**0.698**
Male	414 (31.94)	113 (30.87)	** **
Female	882 (68.06)	253 (69.13)	** **
**Age at diagnosis**			**0.0004**
Mean (±SD)	68.80 (±8.70)	70.70 (±8.50)	** **
Median [Q1—Q3]	69 [62–76]	72 [64–77]	** **
**Age at TKR**			**0.034**
Mean (±SD)	71.11 (±8.52)	72.25 (±8.36)	** **
Median [Q1—Q3]	71 [65–78]	73 [66–78]	** **
**Presence of long-term disease(s) at diagnosis**			**0.177**
Yes	429 (33.10)	135 (36.89)	** **
No	867 (66.90)	231 (63.11)	** **
**Charlson index**			**0.613**
0	491 (37.89)	149 (40.71)	** **
1–2	601 (46.37)	161 (43.99)	** **
≥ 3	204 (15.74)	56 (15.30)	** **
**Primo-prescriber's specialty**			**< 0.0001**
General practice (GP)	241 (18.60)	147 (40.16)	** **
Rheumatology/Physical medicine and rehabilitation	954 (73.61)	202 (55.19)	** **
Orthopedic surgery	101 (7.79)	17 (4.64)	** **
**Number of annual visits to the GP**			**< 0.0001**
Mean (±SD)	9.09 (±5.54)	11.20 (±7.20)	** **
Median [Q1—Q3]	7.91 [5.55–11.61]	10.14 [6.3 -, 14.73]	** **
**At least one hospitalization (TKR excluded)**			**0.001**
Yes	820 (63.27)	197 (53.83)	** **
No	476 (36.73)	169 (46.17)	** **
**Number of annual visits to the specialists of joint diseases** **†**			**< 0.0001**
Mean (±SD)	5.08 (±3.85)	4.55 (±5.38)	** **
Median [Q1—Q3]	4.25 [2.52–6.57]	3.18 [1.24–6.07]	** **
**Number of annual IA HA injections**			**<0.0001**
Mean (±SD)	3.33 (±2.84)	0 (±0)	** **
Median [Q1—Q3]	2.62 [1.58–4.24]	0 [0–0]	** **
**Number of days between inclusion and first IA HA injection(s)**			**NA**
Mean (±SD)	165 (±277)	-	
Median [Q1—Q3]	72 [24–192]	-	
**Number of annual IA CS injections** **‡**			**< 0.0001**
Mean (±SD)	2.00 (±2.21)	3.63 (±5.29)	** **
Median [Q1—Q3]	1.41 [0.74–2.49]	2.21 [1.20–3.86]	** **
**Number of days between inclusion and first IA CS injection(s)** **‡**			**<0.0001**
Mean (±SD)	191 (±332)	82 (±94)	
Median [Q1—Q3]	64 [14–214]	42 [11–130]	
**Number of knees with OA**			**0.0147**
Two	603 (46.53)	144 (39.34)	** **
One/Unknow	693 (53.48)	222 (60.66)	** **
**Number of follow-up days**			**< 0.0001**
Mean (±SD)	864 (±601)	573 (±548)	** **
Median [Q1—Q3]	698 [391–1,224]	367 [192–769]	** **
Range	[49–2,794]	[21–2,493]	** **

No missing data

HA group: patients who received at least one IA HA injection during their follow-up period, N-HA group: patients who received only CS as IA injections, GP: general practionner, TKR: total knee replacement, OA: osteoarthritis, IA: intra-articular, HA: hyaluronic acid, CS: corticosteroids

* n (%) unless stated otherwise

** T-test, Wilcoxon (for mean difference) or Chi-squared tests (for percentage difference) (NA: not applicable)

† Rheumatologist, orthopedic surgeon and physical medicine and rehabilitation practitioner

‡ Calculated on 1,247 Patients who received at least one IA CS injection during their follow-up period (n = 881 for HA group, n = 366 for N-HA group)

**Fig 2:**
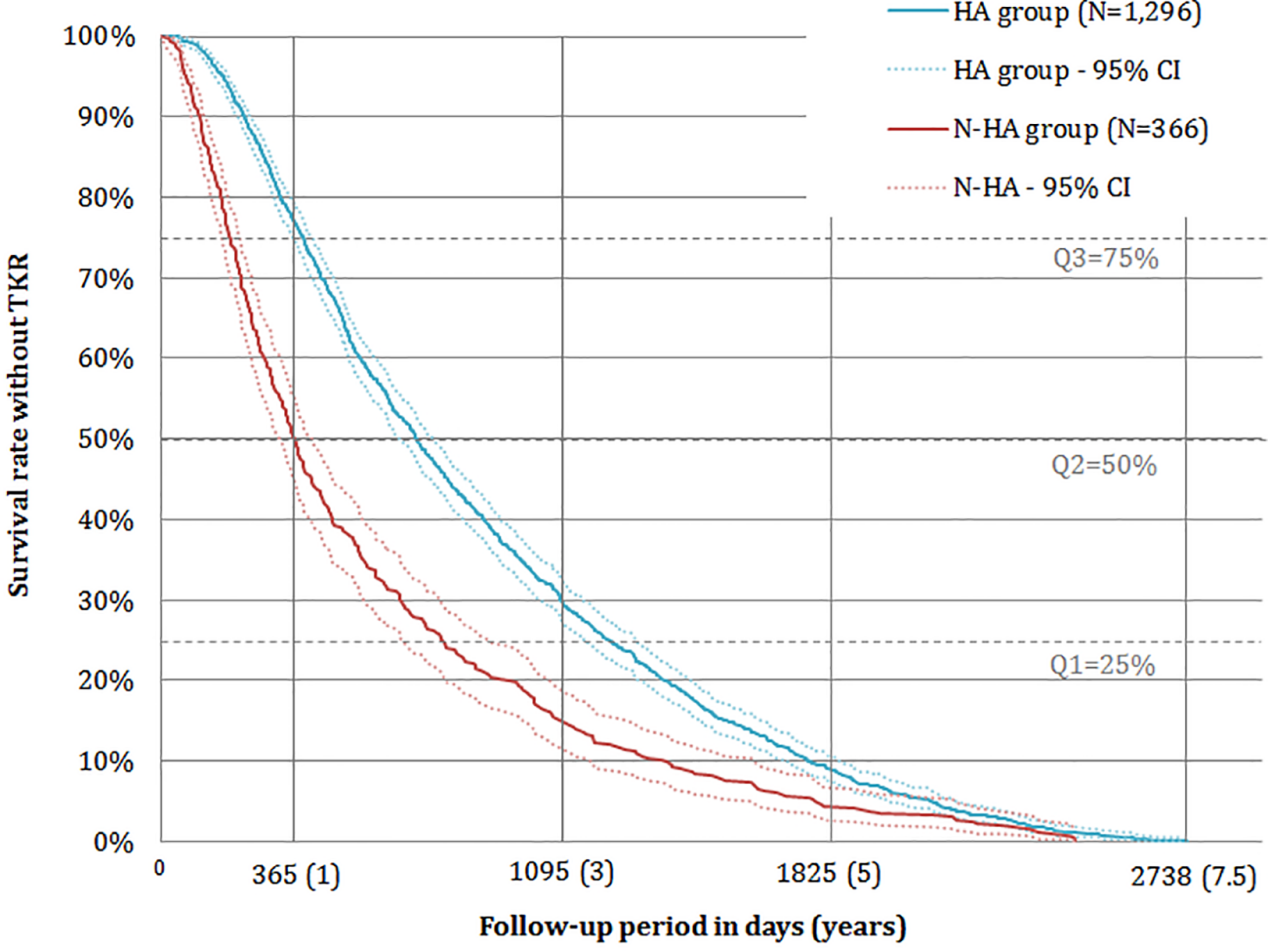
Kaplan-Meier curves of time without TKR for HA and N-HA groups. HA group: patients who received at least one IA HA injection during their follow-up period, N-HA group: patients who received only CS as IA injections, TKR: total knee replacement, IA: intra-articular, HA: hyaluronic acid, CS: corticosteroids.

The KM curves show that the difference in time-to-TKR between treatment groups was present from index date (day 0) to the end of the study (2,794 days) (Fig 2). For each quartile, i.e., 75%, 50% and 25% of patients, the number of days without TKR in the HA group was higher than the number of days without TKR in the N-HA group: 391 days (95%CI [362,413]) *versus* 192 days (95%CI [169,216]), 698 days (95%CI [657,744]) *versus* 367 days (95%CI [323,413]) and 1,224 days (95%CI [1,156,1,294]) *versus* 769 days (95%CI [660,899]) for Q3, Q2 and Q1, respectively. Furthermore, approximately 16% of patients treated with IA HA injections survived more than 1,500 days (4.1 years) without TKR compared with 8% of patients not treated with HA. In addition, the log-rank test rejected the hypothesis of homogeneous KM curves (p-value<0.0001).

RMST are presented in Table 3, i.e., the mean time-to-TKR at each time point that was computed using the stratified Cox model. All differences between groups were significant (Zucker test: p-values<0.0001) in favor of patients treated with IA HA injection(s). Differences in RMST between groups went from 51 (±1) days for 1 year after diagnosis, to 217 (±10) days for 7.5 years after diagnosis. In other words, having at least one IA HA injection increased the mean time-to-TKR by 217 days (7 months) during the 7.5 years following diagnosis of knee OA: 842 days (95%CI [832,852]) for the HA group and 625 days (95%CI [605,646]) for the N-HA group. Table 4 presents significant hazard ratios (HR) for adjustment variables. Women, younger patients, patients with significant comorbidities (high Charlson index/hospitalization) had significantly lower HR for TKR (all p-values<0.05). Regarding the number of annual visits to specialists, all else being equal, having more than 10 specialist visits per year was associated with an instantaneous rate of TKR 7 times greater than when having up to 4 visits per year (p-values<0.0001).

**Table 3:** Restricted mean survival time estimates from the stratified Cox Model.

Time point	RMST HA group [95% CI]		RMST N-HA group [95% CI]		RMST difference (±SD)	Zucker test (p-value)
**365 days (1 year)**	333.18	[332.91, 333.46]	282.35	[280.40, 284.30]	+50.83 (±1.10)	< 0.0001
**1095 days (3 years)**	681.56	[677.93, 685.18]	507.95	[497.87, 518.02]	+173.61 (±5.16)	< 0.0001
**1825 days (5 years)**	807.37	[799.84, 814.90]	592.42	[576.36, 608.49]	+214.94 (±7.95)	< 0.0001
**2794 days (7.5 years)**	842.18	[832.13, 852.23]	625.41	[605.23, 645.60]	+216.77 (±9.70)	< 0.0001

The use of RMST to analyze time-to TKR of patients with knee OA allows testing the difference between treatment groups at different time points from diagnosis. For instance, the difference of RMST at 365 days is about 51 days in favor of HA group, we might say that HA might increase time-to TKR of knee OA patients by 51 days during the first year following diagnosis.RMST: restricted mean survival time, CI: confidence interval, HA group: patients who received at least one IA HA injection during their follow-up period, N-HA group: patients who received only CS as IA injections, TKR: total knee replacement, OA: osteoarthritis, IA: intra-articular, HA: hyaluronic acid, CS: corticosteroids.

**Table 4:** Hazard ratios of adjustment covariates from the stratified Cox model.

		HR*	95% CI*	P-value
***Socio-demographic***	**Gender**	** **	** **	** **
	Male	ref.		** **
	Female	0.883	[0.795, 0.982]	**0.021**
	**Age at diagnosis (continuous)**	1.018	[1.013, 1.024]	**< 0.0001**
***Knee OA condition***	**Primo-prescriber's specialty**	** **	** **	** **
	Rheumatology/Physical medicine and rehabilitation	ref.		** **
	Orthopedic surgery	1.148	[0.948, 1.391]	**0.159**
	General practice (GP)	1.208	[1.068, 1.367]	**0.003**
	Annual visits to the specialists of joint diseases**	** **	** **	** **
	Up to 4 times	ref.		** **
	From 5 to 10 times	2.126	[1.906, 2.372]	**< 0.0001**
	More than 10 times	7.281	[6.066, 8.740]	**< 0.0001**
	**Number of knee with OA**	** **	** **	** **
	Two	ref.		** **
	One/Unknow	1.406	[1.271, 1.555]	**< 0.0001**
***General health condition***	**Presence of long-term disease(s) at diagnosis**	** **	** **	** **
	No	ref.		** **
	Yes	1.218	[1.082, 1.372]	**0.001**
	**Charlson index**	** **	** **	** **
	0	ref.		** **
	1–2	0.848	[0.760, 0.947]	**0.003**
	3 and higher	0.842	[0.712, 0.995]	**0.043**
	**Annual visits to the GP**	** **	** **	** **
	Up to 12 times	ref.		** **
	From 13 to 24 times	1.151	[1.020, 1.298]	**0.022**
	More than 24 times	1.892	[1.396, 2.564]	**< 0.0001**
	**At least one hospitalization (TKR excluded)**	** **	** **	** **
	No	ref.		** **
	Yes	0.489	[0.440, 0.544]	**< 0.0001**

* HR = hazard ratio and CI = confidence interval

** Rheumatologist, orthopedic surgeon and physical medicine and rehabilitation practitioner

GP: general practitioner, TKR: total knee replacement, OA: osteoarthritis.

### Direct medical costs in ambulatory care before TKR

Table 5 summarizes the total direct medical costs per year from 1 year to 7 years before TKR for all patients and according to treatment groups. There was not a significant difference between treatment groups except for the year TKR-5 where costs were higher in the HA group (p-value = 0.04). We highlighted that costs increased as patients got closer in time to arthroplasty in both groups; means of total ambulatory costs increased 8% per year on average. Costs were from €474 (±281) (Q1–Q3 = [296–584]) and €544 (±394) (Q1–Q3 = [278–859]) for the year TKR-7 to €744 (±600) (Q1–Q3 = [371–934]) and €805 (±652) (Q1–Q3 = [402–1,8]) for the year TKR-1 for HA and N-HA groups, respectively.

**Table 5:** Total ambulatory care costs per year from 1 to 7 years before TKR by treatment group.

Total ambulatory care costs (€)		HA group who had TKR	N-HA group who had TKR	P-values*
**year TKR-1**	**n**	**1,296**	**366**	** **
** **	Mean (±SD)	744 (±600)	805 (±652)	**0.104**
** **	Median [Q1—Q3]	597 [371–934]	618 [402–1,8]	** **
**year TKR-2**	**n**	**997**	**184**	** **
** **	Mean (±SD)	661 (±484)	686 (±625)	**0.544**
** **	Median [Q1—Q3]	526 [336–808]	492 [324–812]	** **
**year TKR-3**	**n**	**621**	**97**	** **
** **	Mean (±SD)	615 (±448)	613 (±497)	**0.612**
** **	Median [Q1—Q3]	485 [307–758]	473 [279–759]	** **
**year TKR-4**	**n**	**387**	**55**	** **
** **	Mean (±SD)	592 (±437)	508 (±373)	**0.088**
** **	Median [Q1—Q3]	474 [309–735]	395 [252–665]	** **
**year TKR-5**	**n**	**228**	**31**	** **
** **	Mean (±SD)	571 (±420)	435 (±309)	**0.039**
** **	Median [Q1—Q3]	472 [285–720]	331 [233–553]	** **
**year TKR-6**	**n**	**117**	**16**	** **
** **	Mean (±SD)	528 (±324)	485 (±352)	**0.384**
** **	Median [Q1—Q3]	463 [294–674]	315 [265–687]	** **
**year TKR-7**	**n**	**47**	**10**	** **
** **	Mean (±SD)	474 (±281)	544 (±394)	**0.983**
** **	Median [Q1—Q3]	396 [296–584]	358 [278–859]	** **

No missing data

n: number of patients, TKR: total knee replacement, HA group: patients who received at least one IA HA injection during their follow-up period, N-HA group: patients who received only CS as IA injections, IA: intra-articular, HA: hyaluronic acid, CS: corticosteroids.

* T-test, Wilcoxon (for mean difference)

**Fig 3:**
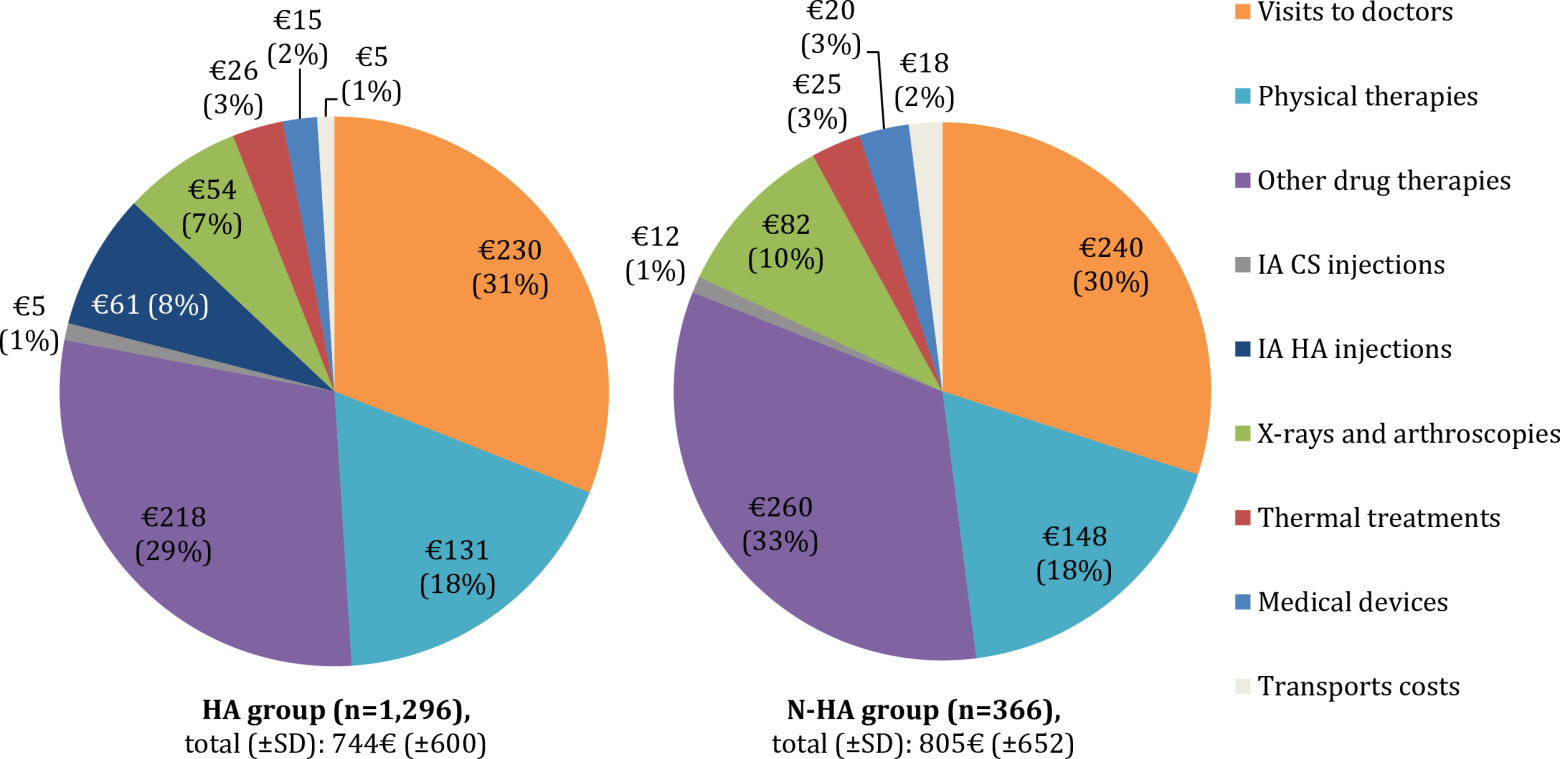
Share of mean of total ambulatory care costs the year before TKR. HA group: patients who received at least one IA HA injection during their follow-up period, N-HA group: patients who received only CS as IA injections, TKR: total knee replacement, IA: intra-articular, HA: hyaluronic acid, CS: corticosteroids, SD: standard deviation. Visits to doctors include visits to rheumatologists, orthopedic surgeons, physicians specialized in physical medicine and rehabilitation or GPs.

Finally, we showed that the share of direct medical costs for TKR-1 was similar between treatment groups (Fig 3). It is noteworthy that the top 3 contributors to the mean of direct medical costs were drug therapies, visits to doctors and physical therapies for HA and N-HA groups, representing 78% and 80% of the mean, respectively. Drug treatment including IA injections (CS and/or HA) corresponded to 38% (€284) for the HA group and 34% (€272) for the N-HA group. Concerning IA injections exclusively, CS represented 1% of costs (approximately €8) in both groups, whereas HA represented 8% of costs (approximately €60) in the HA group.

## Discussion

To our knowledge, this is the first retrospective and observational study on knee OA patients in France to compare time-to-TKR according to the use of IA HA injections. Our findings suggest that HA is associated with a significant delay in TKR. Approximately 7.5 years after diagnosis, patients who received HA had an additional time-to-TKR of 217 (±10) days (0.6 years/7.1 months) on average compared to patients who did not receive HA (p-value<0.0001). Everything else being equal, the RMST was 842 days (95%CI [832,852], 2.3 years) for the HA group and 625 days (95%CI [605,646], 1.7 years) for the N-HA group. In addition, we showed that ambulatory care costs were similar in both groups, i.e., €744 for the HA group and €805 for the N-HA group the year before TKR (p-value = 0.104). These costs included IA injections, which represented approximately €60 (HA and CS) and €8 (CS), respectively.

The main strength of the study is the use of real-world data from an administrative claims database, the EGB, which is a representative sample of the entire insured French population that lends itself to retrospective longitudinal analyses. Given the sheer size of such claims databases, researchers can identify outcomes of patients with rare events more easily, assess the economic impact of various interventions, and gain insight into possible associations between interventions and outcomes [48]. However, the utilization of the EGB was challenged by the lack of clinical information on health outcomes, health status, and symptoms. To address this issue, we selected the study population based on an algorithm developed by the scientific committee of the study. The prevalence rates for knee OA (3.9% for men and 6.9% for women aged 50 years or older) and the TKR rate in this population (11.2% of knee OA patients) were consistent with previous studies. Recent French prevalence rate estimates of symptomatic knee OA are 4.7% for men and 6.6% for women (40–75 years old) [4]. Quintana et al. found that TKR might be appropriate for 15.9% of patients with knee OA [49]. Concerning the cost analysis, while we had the opportunity to work on a comprehensive database, we might have under- or over-estimated some costs. For instance, some oral drugs included in the economic analysis might have been prescribed for diseases other than OA. However, this potential resource allocation bias was similar in both groups and should not impact the study results.

The results of our descriptive analyses are consistent with previous research on knee OA and the use of HA. The mean age was 68 years, and the sex ratio was 2.03 (F:M). A French study involving 10,412 patients with OA reported a sex ratio of 1.96 and a mean age of 66 years [50]. In another French survey involving 607 patients with knee OA aged 40 to 75 years, the sex ratio was 2.41 and the mean age was 62 years [5]. Concerning the analysis of patients who underwent TKR, two recent US studies also using a Cox model approach found similar delays from diagnosis to TKR. Altman et al. retrospectively followed 182,022 knee OA patients with TKR for 6 years and concluded that the use of HA significantly delayed surgery by almost 1 year (median: 484 days for HA users and 114 for others) [37]. Other authors selected 35,146 knee OA patients with TKR from the 5% Medicare sample. Over an 8-year period, HA patients had a significantly longer time–to-TKR compared to other patients; that is, 8.7 extra months (p-value<0.001) [39]. In a discrete event simulation model, Mar et al. showed that HA use was associated with an average delay in the need for surgery of 2.7 years [19]. In these studies, some patients had received both IA HA and IA CS. As was the case in our analysis, the authors did not assess the effect of IA HA on its own but rather the effect of introducing IA HA into the management of patients with OA on the TKR delay. In the literature, the combined effect of IA HA and IA CS showed only an improvement in pain, compared to IA HA alone, limited to the first weeks post IA-CS [51–53]. We estimated the HRs associated with adjustment variables such as gender, age and Charlson index and found that they were similar to those estimated in previous studies [37,39,54], with the exception of the HR associated with the Charlson index in the study by Ong et al. that was not comparable [39]. One explanation for a lower HR for TKR in patients with a high level of comorbidities (Charlson index/hospitalization) could be a balance benefice-risk appreciated negatively by joint disease specialists and/or surgeons (too many contraindications, difficult rehabilitation and limited gains). It is noteworthy that we did not analyze the influence of the HA molecular weight (MW) on the study results. Indeed, because of methodological issues, we could not address this issue in our model; that is, the adjustment covariate HA MW would have been confounding with the group variable (HA and N-HA), thus, the effect of both variables could not be separately identified. Furthermore, the number of patients who were administrated HA with high MW seemed too small to allow for the performance of stratified analyses (175/1,296). However this was not a real issue as the difference in efficacy in terms of pain relief and safety according to the HA MW is still under debate. The ESCEO task force considers that there is currently no clinical evidence supporting an advantage in efficacy of one product over another [55,56].

The mean of total direct medical costs per year for ambulatory care ranged from €474 (±281) to €744 (±600) and €544 (±394) to €805 (±652) in the HA and N-HA groups, respectively, from 7 years to 1 year before TKR. No significant difference was observed between groups except for the year TKR-5 (p-value = 0.04). However, from TKR-5, the number of patients in the N-HA group was small (≤31) compared to the HA group (≤228), which prevented rigorous interpretation of p-values. The increased cost of ambulatory care as patients get closer in time to the TKR is most likely because patients require more medical care to relieve the pain associated with OA, which is particularly high before making the choice of TKR. This was found in a study by Loza et al., which showed that costs increased with clinical and radiologic OA severity [57]. Previous studies have already estimated direct ambulatory costs of knee OA before TKR [10,57–59]. For instance, Le Pen et al. found a mean of €357 per knee OA patient in 2002 in France [10]. In this study, the authors did not mention the date from TKR at which the evaluation was performed and did not take into account the costs of physical therapies or X-rays, which might explain the difference with our estimations. Our results were similar to two European studies, one in Italy and the other in Spain, which found an annual mean of total direct medical costs per knee OA patient of €588 and €677, respectively, when excluding medical help and hospital admissions [57,58]. In the US, a recent paper by Bedard et al. estimated the ambulatory cost of knee OA the year before TKR at $506 (approximately €435) [59]. In contrast to our population in which all patients had used health care before TKR, approximately 34% of their patients had no reimbursement related to knee OA diagnosis the year before TKR, which might explain the difference. Concerning the comparison of direct medical costs between HA and N-HA groups, the fact that there was not a significant difference in total direct medical costs between groups might be the result of fewer prescriptions for oral drugs and X-rays due to IA HA injections. It seems here that additional costs of HA are offset by lower drug expenditures. Mazières et al. showed that a decrease occurred 3 months after IA HA injections and was significant compared to prescription before the injections [60]. Recently, Thomas et al. compared IA HA and NSAIDs groups 6 months before and 6 months after the first HA injection [61] and obtained identical annual expenses; that is, €526 *versus* €528, respectively. These findings were also from the French national health perspective. These amounts seem compatible with those registered during years as early as TKR-6 in our study, and the costs repartition was roughly similar.

However, we must be careful in the interpretation of these results as they do not permit a conclusion in terms of cost effectiveness. It would be interesting to conduct a long-term study that investigate the ability of HA to avoid TKR where hospitalization costs are approximately €8,000 [26], i.e., approximately 10 times higher than our estimation of annual ambulatory care costs. HA could be part of solutions to the increasing rate of TKR in the knee OA population, more specifically, for young patients who are increasing in number and have a higher risk of TKR [22,62].

## Conclusion

The results of this study suggest the effectiveness of HA in delaying TKR in patients with knee OA. In addition, direct medical costs per year before TKR are similar on average in both HA patients and non-HA patients. These results support the benefits of HA and should encourage the implementation of further cost-effectiveness analyses that explore the ability of HA injections to avoid TKR over a longer period of time.

## Supporting information

S1 Table Intra-articular hyaluronic acid (IA HA) injections products used during the study. (DOCX)

S2 Table Intra-articular corticosteroids (IA CS) injections products used during the study. (DOCX)
