# Supplementary material for: Do intra-articular hyaluronic acid injections delay total knee replacement in patients with osteoarthritis – A Cox model analysis
Source: PLoS One. 2017 Nov 20;12(11):e0187227. doi: 10.1371/journal.pone.0187227 (PMC5695798; doi:10.1371/journal.pone.0187227)
Supplement: S1 Table — (DOCX) [file pone.0187227.s001.docx]

S1 Table. Intra-articular hyaluronic acid injections products used during the study.

| **Product (IA HA injection)** | **Hyaluronic acid** | **Molecular weight (10⁶ Da)** | **Concentration (Quantity)** | **No injections per treatment** | **Reimbursement authorization date** |
| --- | --- | --- | --- | --- | --- |
| Arthrum® H 2% | Sodium hyaluronate | [2.2 , 2.6] | 20 mg/ml (2 ml) | 3 | 09/2003 to over 2016 |
| Hyalgan® | Sodium hyaluronate | [0.5 , 0.7] | 10mg/ml (2 ml) | 3 | 10/1992 to over 2016 |
| Adant® | Sodium hyaluronate | [0.6 , 1.2] | 10mg/ml (2,5 ml) | 3 | 09/2003 to over 2016 |
| Go-on® | Sodium hyaluronate | 1.4 | 10mg/ml (2,5 ml) | 3 | 07/2007 to over 2016 |
| Ostenil® | Sodium hyaluronate | 1.2 | 10mg/ml (2 ml) | 3 | 09/2003 to over 2016 |
| Structovial® | Sodium hyaluronate | 1.6 | 10mg/ml (2 ml) | 3 | 09/2005 to over 2016 |
| Suplasyn® | Sodium hyaluronate | [0.5 , 0.75] | 10 mg/ml (2 ml) | 3 | 09/2003 to 09/2010 |
| Synocrom® | Sodium hyaluronate | 1.6 | 10 mg/ml (2 ml) | 3 | 09/2004 to over 2016 |
| Sinovial® | Sodium hyaluronate | [0.8 , 1.2] | 8 mg/ml (2 ml) | 3 | 09/2003 to over 2016 |
| Euflexxa® | Sodium hyaluronate | [2.4 , 3.6] | 10mg/ml (2 ml) | 3 | 03/2008 to over 2016 |
| Orthovisc® | Sodium hyaluronate | [1.0 , 2.9] | 15mg/ml (2 ml) | 3 | 02/2005 to 01/2014 |
| Synvisc® | Hylan G-F 20 | 6 | 8 mg/ml (2 ml) | 3 | 09/2003 to 11/2010 |
| Synvisc-One® | Hylan G-F 20 | 6 | 8 mg/ml (6 ml) | 1 | 02/2009 to over 2016 |
| Viscorneal® | Sodium hyaluronate | 6 | 10mg/ml (2 ml) | 3 | 09/2003 to 09/2010 |
| Durolane® | NASHA | > 90 | 20 mg/ml (3 ml) | 1 | 02/2005 to over 2016 |

10⁶ Da : mega-Dalton

IA: intra-articular, HA: hyaluronic acid, NASHA : non-animal stabilized hyaluronic acid
