# Supplementary material for: Do intra-articular hyaluronic acid injections delay total knee replacement in patients with osteoarthritis – A Cox model analysis
Source: PLoS One. 2017 Nov 20;12(11):e0187227. doi: 10.1371/journal.pone.0187227 (PMC5695798; doi:10.1371/journal.pone.0187227)
Supplement: S2 Table — (DOCX) [file pone.0187227.s002.docx]

S2 Table. Intra-articular corticosteroids (IA CS) injections products used during the study

| **Product (IA CS injection*)** | **International Nonproprietary Name (INN): CS** | **Concentration (Quantity)** | **Reimbursement authorization date** |
| --- | --- | --- | --- |
| Altim® | Cortivazol | 2.5 mg/ml (1.5 ml) | 12/1997 - present |
| Betnesol® | Betamethasone | 4 mg/ml (1 ml) | 11/1997 - present |
| Celestene 4® | Betamethasone | 4 mg/ml (1 ml) | 11/1997 - 12/2013 |
| Celestene Chronodose® | Betamethasone | 5.7 mg/ml (1 ml) | 12/1997 - present |
| Diprostene® | Betamethasone | 7 mg/ml (1 ml) | 03/1998 - present |
| Hexatrione® | Triamcinolone | 20 mg/ml (2 ml) | 12/1997 - present |
| Hydrocortisone Biocodex® | Hydrocortisone | 50 mg/ml (2 ml) | 10/1992 - present |
| Hydrocortisone Upjohn® | Hydrocortisone | 50 mg/ml (2 ml) | 07/1977 - present |
| Depo-medrol® | Methylprednisolone | 40 mg/ml (1 ml) | 11/1997 - 10/2011 |
| Hydrocortancyl® | Prednisolone acetate | 25 mg/ml (5 ml) | 03/1998 - present |
| Kenacort retard® | Triamcinolone | 40 mg/ml (1 ml) | 11/1997 - present |

IA: intra-articular, CS: corticosteroids.
